# Determinants of Major Choice and Academic Expectations: Testing a Prediction Model Across Gender

**DOI:** 10.3389/fpsyg.2022.847843

**Published:** 2022-04-07

**Authors:** Sonia Alfonso, António M. Diniz, Angeles Conde, Mar García-Señorán

**Affiliations:** ^1^Department of Evolutionary Psychology, University of Vigo, Ourense, Spain; ^2^Department of Psychology, Research Center in Education and Psychology, School of Social Sciences, University of Évora, Évora, Portugal

**Keywords:** major choice, academic expectations, first-year students, gender, structural equation modeling

## Abstract

With this study, we aim to test the predictive relationships between determinants of major choice (DMC) and academic expectations (AEs) and to analyze gender differences, using six items of the Determinants of Major Choice Scale and the Academic Perceptions Questionnaire to assess AEs. A convenience sample of Portuguese (*n* = 839) and Spanish (*n* = 1,001) first-year students (age-range = 17–23 years), mostly composed of women (56.9%, *n* = 1,047), was selected from two public universities. The invariance of the multivariate regression model with latent variables of the effect of DMC on AEs, with determinants linked to Personal Characteristics (PCs; e.g., capacities) and Mediating Agents (MAs; e.g., parents) as AE predictors, was tested across gender with LISREL. The invariance test of the multivariate regression model across gender fit the data well and revealed an equivalence of slopes between women and men, which allows a unique interpretation of the model’s predictive relationships for both genders. We also found statistically significant predictive relationships of PCs for six AE factors and MAs for five AE factors. The results showed theoretical relationships with the self-determination theory. At a practical level, they indicated the importance of PCs and MAs to design AE intervention programs in Higher Education (HE) institutions.

## Introduction

Educational expectations in Higher Education (HE) are students’ aspirations and desires about what they hope to achieve during their academic life. Educational expectations are cognitive and motivational, so they represent a key element for adaptation to the university context and the decision to prolong studies (Cabrera et al., 1993; Tinto, 1993; Braxton et al., 2014). They constitute the mental foundations of everyday engagement, which guides student behavior inside and outside educational institutions toward higher-order educational goals such as high school or college graduation (Lorenz et al., 2020). Educational expectations represent the lower or realistic limit of a continuum of educational alternatives ordered from a lower to a higher level of difficulty where aspirations are at the upper or idealistic limit. Along this continuum, students make decisions about educational alternatives based on their potential for fulfillment and their personal value for the students (Zimmermann, 2020). Students adapt and revise their expectations in a continuous Bayesian updating process (Morgan, 2005) in response to new information received or educational experiences. Thus, educational expectations provide early insight into students’ subjective perceptions of opportunities and constraints to further study (Anders, 2017).

The development of the continuum, represented by educational expectations and aspirations, requires time and mental maturity. Children broaden their knowledge of the social structure and their position in it through the family, the peer group, and the school, and later on, as youths, they become aware of their potential, and of the classification of occupations according to their prestige, and they recognize the interaction between income, occupation, and educational level (Gottfredson, 2002). Although the students must evolve to discover their desired place in society and to be able to distinguish between ideal or real aspirations (expectations), youth is where a potentially realizable educational choice is made throughout their educational trajectory (Zimmermann, 2020). These predictions or expectations, which may be more or less idealized or realistic, influence behavior: if it occurs, the planned behavior will be performed in line with the interpretation of the situation. This influence occurs by translating experiences, knowledge, attitudes, motivations, and beliefs into academic actions (Kuh et al., 2005). Understanding one’s subsequent behavior grants an adaptive role to expectations that may or may not match the attainment of students’ desires and hopes.

In the context of HE, a student’s career choice is a crucial moment that will have a high impact on their professional life and future achievements (Ahmed et al., 2017). An appropriate career choice will make the student feel satisfied and motivated, whereas an inappropriate career choice can lead to the abandonment of their studies (Rodríguez-Muñiz et al., 2019). As for the change of major subject, one of the aspects that differentiate students who change from those who do not is that the former attribute less primacy to their interests and skills when choosing a course (Diniz and Almeida, 2007). On another hand, students’ motivation and positive expectations toward studies (educational expectations) are variables that promote academic permanence and success (Castillo-Sánchez et al., 2020).

There are various ways of classifying the determinants of career choice that condition success or failure: individual and contextual (Zacher et al., 2019); internal (personal characteristics, interests: Päßler and Hell, 2012; Zafar, 2013) and external (socioeconomic background: Ma, 2009; Parker et al., 2012). In addition, a third type, interpersonal determinants, is related to the influence of mediating agents (parental support, peer influence, and the interaction with teachers and other educators: Whiston and Keller, 2004; Fouad et al., 2010; Lerkkanen et al., 2012). These classifications are an anchor in the Social Cognitive Career Theory (SCCT; Lent et al., 1994). Among the determinants of career choice, the internal determinants seem to be the most important for the choice of major subjects in both genders (Päßler and Hell, 2012; Zafar, 2013).

From the interaction of the set of internal, external, and contextual factors, students obtain various types of information that consolidate into a series of perceptions about the curriculum of the chosen degree program. These perceptions or “*a priori* expectations” will be modified over time, based on the actual knowledge of the studies acquired while pursuing the degree (Navarro and Soler, 2014), and will determine whether or not the career choice was appropriate (Dias, 2011). Thus, a close relationship between determinants of career choice and educational expectations can be assumed.

Current studies link internal determinants of career choice with aspirations and expectations of job satisfaction, development of autonomy and self-efficacy, and enhancement of learning experiences, in addition to deepening of knowledge in the area of interest (Eren, 2017; McLean et al., 2019). External determinants of career choice relate to aspirations and expectations associated with pursuing a particular profession that ensures appropriate financial remuneration, job security, and promotion opportunities (Akosah-Twumasi et al., 2018). Parents, teachers, and friends also play a relevant role in consolidating college students’ expectations, especially those linked to opportunities for socialization in that context (Akosah-Twumasi et al., 2018; Lorenz et al., 2020) and those related to satisfying the desires of others (Guan et al., 2015).

## Purpose of the Present Study

The main objective of this study is to test a predictive model between determinants of major choice (DMC) and academic expectations (AEs) of first-year university students, analyzing their gender invariance.

From a multifactorial conception of AEs, they were grouped into seven factors related to future employability, personal and social development, student mobility, political/citizen engagement, social pressure, quality of education, and social interaction. These factors were analyzed considering country (Deaño et al., 2015) and country and gender (Diniz et al., 2018), resulting in a cross-cultural validation of a questionnaire to assess them (Almeida et al., 2018), which was used in this study. The DMC considered in this study involves the influence of personal characteristics (PCs) and mediator agents (MAs). We used six items retrieved from the Determinants of Major Choice Scale (Diniz, 2008).

Taking into account the target variables and the instruments used for their operationalization, as a first hypothesis, we expected that their psychometric properties would allow testing a prediction model between the DMC and the factors of expectations. Furthermore, considering the variability and number of factors that affect the DMC and AEs, country and gender were introduced as variables to be considered in the study.

As a second hypothesis, we conjectured that the DMC factors would have a significant impact on the factors of expectations. To our knowledge, no research has considered both types of constructs conjointly through a prediction model while considering gender, a key variable of major selection (Parker et al., 2012). The exploration of the DMC’s impact on AEs through a multivariate regression model with latent variables across gender can shed light on these relationships.

## Materials and Methods

### Sample

A convenience sample of 1,840 Portuguese (*n* = 839) and Spanish (*n* = 1,001) first-year university students (age-range = 17–23 years; *Mdn* = 18) was selected from two public universities. More of the voluntary participants in this study were enrolled in the humanities and social studies area (60.9%, *n* = 1,121) than in the scientific and technological area. Most participants were women (56.9%, *n* = 1,047).

### Materials

We used six items retrieved from two factors of a scale for the assessment of HE students’ valuation of the DMC (Diniz, 2008): determinants linked to PCs (e.g., capacities) and MAs (e.g., parents). Items are rated on a 5-point Likert-type scale, ranging from 1 (*not at all important*) to 5 (*decisive or extremely important*).

To assess AEs, we used the Academic Perceptions Questionnaire (APQ; Almeida et al., 2018) which measures seven factors of expectations, with six items each: (1) Training for Employment (TE), (2) Personal and Social Development (PSD), (3) Student Mobility (SM), (4) Political Engagement and Citizenship (PEC), (5) Social Pressure (PS), (6) Quality of Education (QE), and (7) Social Interaction (SI). Items are rated on a 6-point Likert-type scale ranging from 1 (*totally disagree*) to 6 (*totally agree*).

### Data Collection and Analysis

We collected the data before the SARS-CoV-2 pandemic, at the beginning of the first semester (mid-October and the beginning of November), after obtaining students’ informed consent. We used the IBM SPSS Statistics for Windows (version 24.0) for descriptive data analysis and to deal with missing values (substituted by the respective distributional median).

The invariance of the multivariate regression model with latent variables, or factors, of the effect of DMC on AEs, with both PCs and MAs as AE predictors, was tested across gender with LISREL 8.80 (Jöreskog and Sörbom, 2006).

We performed the multivariate regression model invariance test across gender only after the invariance testing, through confirmatory factor analysis, of its analogous 9-factor oblique model across countries and gender, and the subsequent inspection of its psychometric properties to complete the model’s structural validity study (Anderson and Gerbing, 1988; Jöreskog and Sörbom, 1993). We followed Fornell and Larcker (1981) criteria of factors’ convergent and discriminant validity (CV and DV), as well as their composite reliability (CR). CV is based on the items’ average variance extracted (AVE), which should be at least 0.50, and DV is based on the comparisons of any two factors’ shared variance (φ^2^; squared disattenuated correlation) and the AVE of each factor, which should be higher than φ^2^. Factors’ reliability should be at least 0.70, and 0.80 is desirable for group comparisons (Nunnally and Bernstein, 1994).

Because the observed variables were ordinal, model estimation and testing were performed with the underlying bivariate normal approach (Jöreskog, 2005), using the robust Satorra–Bentler (SB) scaled correction for maximum likelihood (Satorra and Bentler, 1994). This approach involves the estimation in PRELIS 2 (Jöreskog and Sörbom, 1996) of the means and the asymptotic covariance matrix of the polychoric covariances of each group’s latent normal counterparts of the observed variables, under thresholds fixed to the pooled thresholds estimated in the combined group. The result of this multi-group analysis was used as input for model estimation and testing with the SIMPLIS command language (Jöreskog and Sörbom, 1993) under the independence of the items’ error measurement, or residuals (uniqueness and random error), and the factor’s identification was ensured by setting to one (1.00) the path to one of its items.

The analysis was conducted by comparing the 9-factor oblique model’s form of invariance (all parameters freely estimated across groups) with a more restrictive model (i.e., with more degrees of freedom), the fully invariant model, which is invariant across groups at measurement (factor scores, intercepts, and residuals) and factor levels (variances and covariances).

The multivariate regression model was specified by freely estimating the error covariances between the criteria (AE factors), assuming that the predictors (PCs and MAs) do not capture the totality of their correlations. The model was tested across gender with different slopes and then, with equal slopes (Jöreskog and Sörbom, 1993) to assess the invariance of the model with equal slopes.

The assessment of model invariance was based on the variation (Δ) of the comparative fit index (CFI) and, in addition, on the following goodness-of-fit indices and recommended benchmarks to indicate a good fit (Hu and Bentler, 1998): a comparative fit index (CFI) close to or above 0.95, a root mean square error of approximation (RMSEA) close to or below 0.06, and a standardized root mean square residual (SRMR) close to or below 0.08. Values of δCFI between a restricted model and a baseline model of less than −0.01 indicate non-invariance of the restricted model (Cheung and Rensvold, 2002).

## Results

### Oblique Model Invariance Across Countries and Gender

The 9-factor oblique model was fully invariant across countries and gender, as shown in Table 1, but with the nuance of a differential item functioning between countries presented by Item 37 of the APQ. This difference occurred in its intercepts (Spain = 5.61; Portugal = 3.53), not indicating different levels of item ambiguity but merely differences in the item’s attractiveness to the samples (Ferrando, 1996).

**TABLE 1:** 9-Factor oblique model: factorial invariance across countries and gender.

Model	SBχ ^2^_(*df*)_	RMSEA_[90% CI]_	SRMR		CFI
			
*Countries*			Spain(_*n* = 1,001_)	Portugal(_*n* = 839_)	
M1	7314.14_(2,088)_	0.052_[0.051–0.054]_	0.075	0.077	0.975
M2	9846.39_(2,268)_	0.060_[0.059–0.062]_	0.101	0.094	0.963
M3	9284.72_(2,267)_	0.058_[0.057–0.060]_	0.101	0.095	0.966
M1-M3					ΔCFI = −0.009

***Gender***			**Men(_*n* = 793_)**	**Women(_*n* = 1,047_)**	

M1	7007.23_(2,088)_	0.051_[0.049–0.052]_	0.070	0.076	0.974
M2	7974.66_(2,268_)	0.052_[0.051–0.054]_	0.099	0.079	0.970
M1-M2					ΔCFI = −0.006

*M1, form invariance; M2, M1 fully invariant; M3, M2 with the intercept of Item 37 of the Academic Perceptions Questionnaire freely estimated across countries (Spain 5.61, Portugal, 3.53). RMSEA, root mean square error of approximation; SRMR, standardized root mean square residua; CFI, comparative fit index; Δ = difference between tested model and baseline model.*

In Tables 2, 3, we present the psychometric data for the fully invariant 9-factor oblique model by country and gender.

**TABLE 2:** Fully invariant 9-factor oblique model by country and gender: common metric’s robust maximum likelihood estimates completely standardized, convergent validity, and composite reliability (*N* = 1,840).

	Countries		Gender	
		
Item (Factor)	β	*R* ^2^	β	*R* ^2^
Personal aptitudes and capacities (PCs)	0.71	0.50	0.70	0.49
Interest in the professional area	0.67	0.45	0.68	0.46
Way of being and personal characteristics	0.74	0.54	0.73	0.54
AVE/CR	0.50/0.75		0.50/0.75	
Parents, siblings, or other relatives (MAs)	0.71	0.50	0.70	0.50
Friends, colleagues, or girlfriend/boyfriend	0.70	0.50	0.71	0.50
Teachers	0.53	0.28	0.54	0.29
AVE/CR	0.43/0.69		0.43/0.69	
1. Achieve a prestigious profession (TE)	0.59	0.35	0.57	0.32
8. Have better career opportunities in the job market	0.79	0.62	0.77	0.59
15. Obtain training to achieve a good job	0.83	0.69	0.83	0.69
22. Qualify to achieve professional success	0.84	0.71	0.84	0.71
29. Ensure a successful professional career	0.82	0.67	0.83	0.69
36. Achieve in-service training to facilitate access to work	72	0.52	0.72	0.52
AVE/CR	0.59/0.90		0.58/0.89	
2. Improve my identity, autonomy, and self-confidence (PSD)	0.69	0.48	0.68	0.46
9. Develop my personality traits	0.72	0.52	0.72	0.52
16. Gain self-confidence in my potential	0.76	0.58	0.76	0.58
23. Have goals in life	0.74	0.55	0.73	0.53
30. Deal autonomously with life’s difficulties	0.74	0.55	0.73	0.53
37. Acquire skills to be a responsible adult	0.77	0.59	0.73	0.53
AVE/CR	0.54/0.88		0.52/0.87	
3. Participate in student exchange programs (SM)	0.80	0.64	0.80	0.64
10. Accomplish a stay in another country	0.84	0.71	0.84	0.71
17. Obtain training that allows me to achieve international employment	0.77	0.59	0.76	0.58
24. Obtain international quality training	0.69	0.48	0.73	0.53
31. Spend some of my study time in another country	0.87	0.76	0.73	0.53
38. Achieve an international title	0.86	0.74	0.77	0.59
AVE/CR	0.65/0.92		0.60/0.90	
4. Contribute to improving the world and society (PEC)	0.77	0.59	0.77	0.59
11. Solve problems that disadvantaged people face	0.77	0.59	0.77	0.59
18. Develop a critical view of the world	0.71	0.50	0.71	0.50
25. Participate in volunteer activities	0.68	0.46	0.68	0.46
32. Be an educated citizen committed to society	0.75	0.56	0.75	0.56
39. Contribute to the improvement of the human condition	0.83	0.69	0.82	0.67
AVE/CR	0.57/0.89		0.56/0.89	
5. Meet my family’s expectations (SP)	0.77	0.59	0.77	0.59
12. Not obtain worse grades than other classmates	0.55	0.30	0.55	0.30
19. Not disappoint my family or friends because of my grades	0.83	0.69	0.83	0.69
26. Seize the educational opportunity provided by my family	0.54	0.29	0.54	0.29
33. Fulfill the desire of people close to me who encourage my higher education	0.75	0.56	0.75	0.56
40. Achieve a close to or higher level of education than that obtained by my parents (or older siblings)	0.57	0.32	0.57	0.32
AVE/CR	0.46/0.83		0.46/0.83	
6. Participate in debates or scientific conferences (QE)	0.52	0.27	0.53	0.28
13. Deepen my knowledge of specific subjects	0.61	0.37	0.62	0.38
20. Participate in research projects	0.53	0.28	0.53	0.28
27. Correspond to society’s investment in higher education	0.65	0.42	0.64	0.41
34. To get a satisfactory academic performance to conform a good curriculum	0.71	0.50	0.72	0.52
41. To have teachers with recognized capacity in the area of training they teach	0.60	0.36	0.59	0.35
AVE/CR	0.37/0.78		0.37/0.78	
7. Enjoy living with others and having fun (**SI**)	0.75	0.56	0.74	0.55
14. Engage in extracurricular activities	0.50	0.25	0.51	0.26
21. Establish a weekly schedule that allows for other activities	0.63	0.40	0.62	0.38
28. Attend university student parties	0.65	0.42	0.65	0.42
35. Have a group of friends with whom I can relax and socialize outside of class	0.85	0.72	0.85	0.72
42. Socializing/connecting with a new group of friends	0.77	0.59	0.77	0.59
AVE/CR	0.49/0.85		0.49/0.85	

*PCs, Personal Characteristics; MAs, Mediating Agents; TE, Training for Employment; PSD, Personal and Social Development; SM, Student Mobility; PEC, Political Engagement and Citizenship; SP, Social Pressure; QE, Quality of Education; SI, Social Interaction. β, standardized factor loading; R^2^ (communality), 1—ε (standardized residual); AVE, average variance extracted; CR, composite reliability.*

**TABLE 3:** Fully invariant 9-factor oblique model by country and gender: Disattenuated correlations from the common metric completely standardized solution (*N* = 1,840).

	PCs	MAs	TE	PSD	SM	PEC	SP	QE	SI
**Countries**									
PCs	1.00								
MAs	0.06	1.00							
TE	0.31	0.06	1.00						
PSD	0.38	0.07	0.78	1.00					
SM	0.16	0.06	0.41	0.48	1.00				
PEC	0.38	0.07	0.51	0.74	0.54	1.00			
SP	0.05	0.41	0.48	0.46	0.29	0.36	1.00		
QE	0.35	0.14	0.80	0.82	0.52	0.78	0.66	1.00	
SI	0.16	0.10	0.57	0.65	0.45	0.48	0.42	0.58	1.00
**Gender**									
PCs	1.00								
MAs	0.06	1.00							
TE	0.33	0.06	1.00						
PSD	0.40	0.07	0.79	1.00					
SM	0.17	0.07	0.41	0.47	1.00				
PEC	0.38	0.07	0.53	0.74	0.53	1.00			
SP	0.00	0.41	0.49	0.46	0.29	0.36	1.00		
QE	0.35	0.15	0.82	0.82	0.52	0.78	0.66	1.00	
SI	0.16	0.12	0.56	0.64	0.45	0.47	0.43	0.58	1.00

*Refer to Table 2 for abbreviations.*

In Table 2, it can be seen that all items represented their respective factors well (β > 0.50), and also that the factors’ CV (AVE) and CR ranged between acceptable to good, except for MAs and, mainly, for QE. The QE factor showed similar problems with other Portuguese and Spanish samples (Diniz et al., 2018). Without Items 6 and 20, the factor CV improves and, in the opposite, its reliability deteriorates in both samples (VME = 0.41; FC = 0.73). Considering previous psychometric results (Diniz et al., 2018), and that the items showed standardized factor loadings higher than 0.50 (all *R*^2^ > 0.26, high effect size; Cohen, 1988), they were retained in the model for further analysis. Furthermore, the SM factor presented a better CV in countries than in gender.

Regarding factors’ DV, taking Tables 2, 3 conjointly, undesirable correlations (φ; Table 3) and, consequently, shared variances (φ^2^), were found between TE–QE (countries, φ^2^ = 0.64; gender, φ2 = 0.67), PSD–QE (countries and gender, φ^2^ = 0.67), and PEC–QE (countries and gender, φ^2^ = 0.61), jeopardizing their DV (see respective CV in Table 2). Similar results for these factors emerged in a previous study (Almeida et al., 2018). In the current study, problems in DV also appeared between TE–PSD (countries, φ^2^ = 0.61; gender, φ^2^ = 0.62), and minor problems between PSD and PEC because their shared variance (countries and gender, φ^2^ = 0.55) was close to and lower than their AVE (see Table 2).

Finally, PC and MA factors were independent (φ = 0.06), verifying the assumption regarding the predictors of the regression model’s gender invariance.

### Regression Model Invariance Across Gender

Once the oblique model’s full invariance was guaranteed across countries and gender, the regression model could be estimated and tested across gender under full invariance at measurement and factor levels to examine the slopes’ invariance between predictors (PCs and MAs) and criteria (AEs).

Before examining the model slopes’ invariance, we noted that model testing revealed a different pattern for PCs and MAs factors’ mean comparisons by gender. Although there were gender differences in PCs, there were no differences in MAs (Table 4).

**TABLE 4:** Comparisons of the means of the model’s predictor factors by gender.

	Men_(*n* = 739)_	Women_(*n* = 1,047)_		
	
Predictor factors	*M*	*M*	*SE*	*T*
Personal characteristics	0.00	0.41	0.07	5.86***
Mediating agents	0.00	−0.05	0.05	−1.02

***p < 0.001.

In Table 4, it can be seen that women presented a significantly higher PC factor means than men, considering that LISREL 8 fixes to zero the factor means of the first group of data, the men’s group, and estimates factor means of the second group of data, the women’s group (Jöreskog and Sörbom, 1993).

**FIGURE 1:**
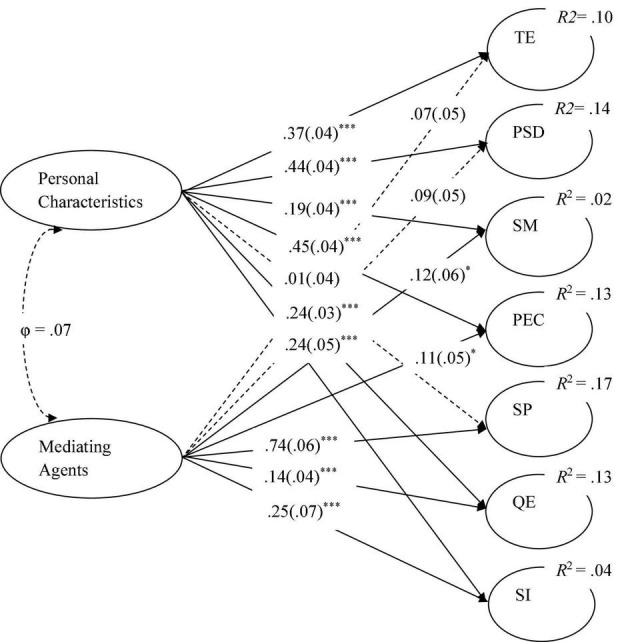
Regression model of the effect of determinants of major choice on academic expectations with equal slopes across gender. Unstandardized robust maximum likelihood estimates for structural relationships (*N* = 1,840). Refer to Table 1 for abbreviations. φ, disattenuated correlation. Standard errors are in parentheses. Dashed arrows, non-significant paths. *p < 0.05. ***p < 0.001.

The regression model with equal slopes was fully invariant across gender (Table 5), and its unstandardized structural estimates mostly presented very significant effects of PCs and MAs on AEs (Figure 1).

**TABLE 5:** Regression model of the effect of determinants of major choice on academic expectations: invariance across gender.

		RMSEA_[90% CI]_	SRMR		CFI
			
Model	SBχ ^2^_(*df*)_		Men_(*n* = 793)_	Women_(*n* = 1,047)_	
M1	7749.08_(2,045)_	0.052_[0.050–0.053]_	0.092	0.076	0.971
M2	7802.73_(2,259)_	0.052_[0.050–0.053]_	0.098	0.078	0.971
M1-M2					ΔCFI = 0.000

*Regression models were estimated and tested under full invariance at measurement and factor levels and with factor correlations of academic expectations freely estimated. M1, model with different slopes; M2, model with equal slopes. Refer to Table 1 for other abbreviations.*

Figure 1 shows that the only AE not well predicted by PCs was SP, and MAs did not predict TE and PSD and only slightly predicted SM. Finally, the magnitude of the effect size of both PCs and MAs on AEs, according to Cohen’s criteria (1988), was around *small* (*R*^2^ = 0.02) for SM and SI, and around *medium* (*R*^2^ = 0.13) for all the others.

## Discussion and Conclusion

With this study, we intended to examine the predictive relationships between DMC and AEs of first-year university students across gender. To pursue this goal, we tested a multivariate regression model of the effect of PC and MA, as determinants of major choice (DMC), on APQ’s 7-factors AEs with large samples of Spanish and Portuguese students.

First, we wanted to determine the model’s possible equivalence across countries and gender and whether its factors would have adequate psychometric properties by country and gender. Similarly, in a two-step approach (Anderson and Gerbing, 1988; Jöreskog and Sörbom, 1993), before testing this regression model, we tested its oblique model counterpart, which was well fitted and denoted full invariance by country and gender, with all the nine factors presenting acceptable psychometric properties (Fornell and Larcker, 1981; Nunnally and Bernstein, 1994). In this process, we also observed that the two model predictors (PCs and MAs) were independent, which allows a clear interpretation of their relationships with the criteria (AEs). Concerning the gender differences in the factor means of the model predictors, we found that women attribute more importance to the PCs for a major choice than men, similar to Nadelson et al. (2013) results. By contrast, the importance of MAs for a major choice does not differentiate between women and men, contradicting the belief that women attribute more importance to MAs than men do (e.g., Päßler and Hell, 2012; Sojkin et al., 2012). This result can be a by-product of the lack of importance attributed by college students to external influences in their decision to pursue HE (Nadelson et al., 2013), reflected in the tendency for career choices in women to be increasingly dispersed and more similar to those of men (Montmarquette et al., 2002; Ma, 2009; Chang and ChangTzeng, 2020). Second, we conjectured that DMC would have a significant impact on the factors of expectations across gender. The invariance test of the multivariate regression model across gender indicated that it was well-fitted to data and revealed an equivalence of slopes (Jöreskog and Sörbom, 1993) between women and men. As a consequence, a unique interpretation of its predictive relationships for both genders is allowed. Traditionally and currently, numerous studies corroborate gender differences in Science, Technology, Engineering, and Mathematics (STEM) students related to career choice motives and students’ educational expectations (Su and Rounds, 2015; Trusz, 2020). Nevertheless, we can speculate that this equivalence of slopes between genders occurs in our study because most of the sample is made up of students from degrees belonging to the social sciences and humanities (60%). On the other hand, it seems that family background and previous learning experiences, among others, play a much more important role in career choice and the formation of AEs than gender (Babarović, 2021). Moreover, educational institutions’ increasing efforts to homogenize formative programs and methodologies minimize gender inequalities (Babarović, 2021; World Economic Forum, 2021).

The equivalent slopes for gender revealed statistically significant predictive relationships of PCs and MAs on six and five of the seven AEs, respectively. Consistent with other studies, we found that PCs predicted AEs related to PEC, PSD (Nyamwange, 2016; Eren, 2017; McLean et al., 2019), and TE (Akosah-Twumasi et al., 2018) more strongly than those related to QE, SI, and SM. Furthermore, PCs did not predict SP, implying that accomplishing the expectations of others or finishing the degree within a given time frame seems to take a back seat to the primacy of students’ self-interests (Brahm et al., 2017).

Along with evidence from some other studies, we also found that MAs’ prediction of AEs, compared to PCs, was higher in SP (Guan et al., 2015) and SI (Akosah-Twumasi et al., 2018; Lorenz et al., 2020) and lower in PEC and SM. No relationship was found between TE and PSD. This may be because the content of the items of these EAs, as presented in the APQ, refer to personal evaluations of access to a job (good job, professional success) and PCs (autonomy, self-confidence). TE and PSD were better predicted by PCs, as in other studies (Navarro and Soler, 2014; Rodríguez-Muñiz et al., 2019).

Overall, we can state, as an educated guess, that students who choose HE studies according to their PCs form AEs based on the possibility of performing experiences and activities in that environment that give them the opportunity to help others to improve and enhance personal skills and achieve a successful professional future. On the other hand, students who base their choice of HE studies more on the influence of MAs expect to find experiences of social interaction in HE and to achieve an academic performance consistent with others’ expectations.

Some theoretical implications can be derived from the findings of this study. First, as far as we know, there are no studies in which the DMC and AEs have been explored conjointly across gender. The multivariate regression model studied offers the opportunity to link these two key elements for academic persistence and success in HE (Bargmann et al., 2021; Eren & Rakıcıoğlu-Söylemez, 2021). It also allows interpretations in tune with Tinto (1993) attrition theory and with those made in the self-determination theory’s studies on the importance of personal value-interest and expectations in career choice and student retention (Duchatelet and Donche, 2019; Eccles and Wigfield, 2020; Ryan and Deci, 2020; Schnettler et al., 2020).

Moreover, within our attempt to link career and academic psychological constructs, the finding that the DMCs’ effect on AEs is equivalent across gender points to an indistinct gender role in the influence of personal and contextual variables of career choice on AEs among first-year university students.

On a practical level, educational institutions could adjust their intervention programs to favor the retention and permanence of students (Bergmark et al., 2018) who have chosen a career to some degree based on their PCs or MAs, knowing what each group expects from HE. Moreover, based on the effect size results, the majority of the effects of PCs and MAs on AEs were of *medium* size, indicating that these two DMCs are relevant in practical terms when intervening on AEs. We also note that, according to study results, this DMC relevance is independent of gender. However, it seems that no practical implications of PCs and MAs on SM and SI should be considered, as *small* effect sizes emerged for these relationships. First-year students vaguely represent these AEs because of their lack of knowledge of the new organizational structure and the opportunities to achieve them in their HE studies (Trautwein and Bosse, 2017). In contrast, we can conjecture that they are a kind of AEs that neither the students nor their significant social environment found relevant for the choice and development of an HE career.

These types of studies can help HE institutions to be more attentive to the students they welcome, to create the necessary conditions for them to have experiences matching their interests and expectations, identify and model adequately planned behaviors that promote academic adaptation and persistence (Dewberry and Jackson, 2018).

Despite the cautions to avoid threats to the study’s internal validity, for example, model estimation and testing using the underlying bivariate normal approach (Jöreskog, 2005) with the robust Satorra and Bentler (1994) correction, and ensuring the model’s psychometric assumptions, the external validity of the statistical conclusions presented is limited, given the non-probabilistic sampling used (Shadish et al., 2002). For example, the replication of the current study in other samples could shed light on the possible generalization of its findings. Also, it would be interesting to extend the obtained results by analyzing the invariance of the model according to the students’ area or their major subject of study, or diverse cohorts of students, e.g., traditional vs. non-traditional students, or first-generation vs. continuing-generation students. We could also conduct longitudinal studies to explore how the model would behave considering the academic organization by semesters or academic years. These studies would help to better customize the interventions to the diversity of subpopulations of HE students.

Finally, this research is a contribution to the study of the significant relationships between the determinants of career choice and academic expectations, factors that are understood as essential both for career development and student retention in HE. In addition, an integrated view of these variables is offered, considering others of a personal and contextual nature, such as gender and nationality.

## Data Availability Statement

The raw data supporting the conclusions of this article will be made available by the authors, without undue reservation.

## Ethics Statement

The studies involving human participants were reviewed and approved by the Ethics Committee of the Ph.D. Program in Education and Behavioral Sciences, University of Vigo. The patients/participants provided their written informed consent to participate in this study.

## Author Contributions

SA, AD, AC, and MG-S: literature review, material preparation, and results’ interpretation. AC, MG-S, and SA: data collection. AD and SA: data analysis and writing of the manuscript. All authors read and approved the final manuscript.

## Conflict of Interest

The authors declare that the research was conducted in the absence of any commercial or financial relationships that could be construed as a potential conflict of interest.

## Publisher’s Note

All claims expressed in this article are solely those of the authors and do not necessarily represent those of their affiliated organizations, or those of the publisher, the editors and the reviewers. Any product that may be evaluated in this article, or claim that may be made by its manufacturer, is not guaranteed or endorsed by the publisher.
